# P226: An engaged intervention to decrease central line-asssociated infections in the ICU

**DOI:** 10.1186/2047-2994-2-S1-P226

**Published:** 2013-06-20

**Authors:** BR Oh, JS Song, EH Choi, HK Noh, PG Choe

**Affiliations:** 1 Infection Control Office, Seoul National University Hospital, SeouL, Korea, Republic Of; 2 Nursing Department, Seoul National University Hospital, SeouL, Korea, Republic Of; 3 Medical Intensive Care Unit, Seoul National University Hospital, SeouL, Korea, Republic Of; 4 Division of Infectious Diseases, Seoul National University Hospital, SeouL, Korea, Republic Of

## Introduction

The infection control guidelines for reducing central line-associated bloodstream infections (CLABSIs) are evidence-based medicine, yet translating evidence into practice (TEIP) is very difficult issue in workplace.

## Objectives

The goal of this intervention is to improve health care workers (HCWs)'s infection control behavior compliance for reducing CLABSIs in a medical ICU of a university hospital in Korea.

## Methods

This intervention was conducted in a unit, medical intensive care unit (22beds) in a university hospital for 11 months. An evidence-based intervention was used to get involved HCWs. 3 times web-based surveillance tests were to observe HCWs' CLABSI guideline knowledge before (pre test) and after( 2nd, 3rd post test). Physicians and Registered Nurses group focusing educations were conducted each group every month. Unit charge nurse checked Central line insertion checklist with 21 points per insertion and central line maintenance checklist with 14 points everyday for 9 months. Rates of infection per 1000 catheter-days were measured by monthly. Analyzed data were shared HCWs.

## Results

A total 174 HCWs reported knowledge test. The level of knowledge after intervention increased from 15.9 at baseline to 18.3 at 6 months after implementation of the study intervention. Central line insertion guideline compliance increased from 2.7% at baseline to 53.3% at 8 months after implementation of the intervention (P<0.001), especially Hand hygiene rate was the most improved from 32.4% to 80.0%(p<=0.002).

## Conclusion

The HCWs involved intervention resulted in a large enlargement infection control behavior compliance. Despite slight decrease of the CLABSIs rate, the effect will be expected later. To reduce CLABSIs, we encourage HCWs behaviors continuously and should consider additional barrier to compliance.

## Disclosure of interest

None declared

